# A study protocol to assess the feasibility of conducting an evaluation trial of the ADVANCE integrated intervention to address both substance use and intimate partner abuse perpetration to men in substance use treatment

**DOI:** 10.1186/s40814-020-00580-7

**Published:** 2020-05-11

**Authors:** Gail Gilchrist, Sabine Landau, Polly Radcliffe, Mary McMurran, Gene Feder, Caroline Easton, Steve Parrott, Sara Kirkpatrick, Juliet Henderson, Laura Potts, Danielle Stephens-Lewis, Amy Johnson, Beverly Love, Gemma Halliwell, Sandi Dheensa, Cassandra Berbary, Jinshuo Li, John Strang, Elizabeth Gilchrist

**Affiliations:** 1 National Addiction Centre, Institute of Psychiatry, Psychology and Neuroscience, King’s College London, 4 Windsor Walk, London, SE5 8BB UK; 2 Department of Biostatistics and Health Informatics, Institute of Psychiatry, Psychology & Neuroscience, King’s College London, De Crespigny Park, London, SE5 8AF UK; 3 Independent Consulting Psychologist, Lancaster, UK; 4 School of Social and Community Medicine, University of Bristol, Canynge Hall, 39 Whatley Road, Bristol, BS8 2PS UK; 5 Rochester Institute of Technology, 153 Lomb Memorial Drive, Rochester, NY 14623 USA; 6 Department of Health Sciences, University of York, Seebohm Rowntree Building, Heslington, York, YO10 5DD UK; 7 RESPECT, The Green House, 244-254 Cambridge Heath Road, London, E2 9DA UK; 8 Centre for Violence Prevention, University of Worcester, Worcester, UK; 9 School of Health in Social Science, University of Edinburgh, 8-9 Hope Park Square, Edinburgh, 8HQ 9NW UK

## Abstract

**Background:**

Strong evidence exists that substance use is a contributory risk factor for intimate partner abuse (IPA) perpetration. Men in substance use treatment are more likely to perpetrate IPA than men from the general population. Despite this, referral pathways are lacking for this group. This trial will assess the feasibility of conducting an evaluation trial of a tailored integrated intervention to address substance use and IPA perpetration to men in substance use treatment.

**Methods/design:**

ADVANCE is a multicentre, parallel-group individually randomised controlled feasibility trial, with a nested formative evaluation, comparing an integrated intervention to reduce IPA + substance use treatment as usual (TAU) to TAU only. One hundred and eight men who have perpetrated IPA in the past 12 months from community substance use treatment in London, the West Midlands, and the South West will be recruited. ADVANCE is a manualised intervention comprising 2–4 individual sessions (2 compulsory) with a keyworker to set goals, develop a personal safety plan and increase motivation and readiness, followed by a 12-session weekly group intervention delivered in substance use services. Men will be randomly allocated (ratio 1:1) to receive the ADVANCE intervention + TAU or TAU only. Men’s female (ex) partners will be invited to provide outcome data and offered support from integrated safety services (ISS). Regular case management meetings between substance use and ISS will manage risk. Outcome measures will be obtained at the end of the intervention (approximately 4 months post-randomisation) for all male and female participants. The main objective of this feasibility trial is to estimate parameters required for planning a definitive trial including rates of consent, recruitment, and follow-up by site and group allocation. Nested formative evaluation including focus groups and in-depth interviews will explore the intervention’s acceptability to participants, group facilitators, keyworkers and ISS workers. Secondary outcomes include substance use, IPA, mental health, self-management, health and social care service use, criminal justice contacts, and quality of life.

**Discussion:**

Findings from this feasibility trial will inform the design of a multicentre randomised controlled trial evaluating the efficacy and cost-effectiveness of the ADVANCE intervention for reducing IPA and improving the well-being of female (ex)partners.

**Trial registration:**

ISRCTN79435190.

## Background

Intimate partner abuse (IPA) includes any incident/pattern of incidents of controlling, coercive or threatening behaviour, violence, or abuse between current or former partners [1]. Women are more likely to experience sexual violence, injury, or be murdered by a partner than men [2–5]. While no single factor explains why some people perpetrate IPA [6, 7], there is strong and consistent evidence that substances, particularly alcohol, cocaine, and methamphetamines are contributory risk factors for IPA perpetration [8–16]. This association is significantly stronger from men who abuse or are dependent on alcohol or drugs [16]. The complex interplay between substance use and IPA perpetration includes intoxication (change or disinhibition when under the influence of alcohol or stimulant drugs), withdrawal and craving (irritability and frustration or the need to acquire substances), the impact of substance use on relationships, the wider dynamics of power and control, and psychological vulnerabilities (adverse childhood experiences, mental health, and emotional instability) linked to substance use and IPA [17, 18]. At least 3 in 10 men receiving substance use treatment have been physically or sexually violent, and 7 in 10 men have been psychologically abusive towards their partner in the previous year [3, 8, 9, 19–21]. These rates are far higher than amongst the general population [12, 14]. Around three-quarters of men attending substance use treatment in England had ever perpetrated any IPA towards their partner [9].

Despite the association between IPA perpetration and substance use, and the higher prevalence of IPA perpetration amongst men receiving substance use treatment, there is a lack of care pathways including perpetrator programmes for this client group [22]. Community perpetrator programmes in England (UK) meet only 10% of the existing demand [23]. Men who use substances are rarely referred to perpetrator programmes and when they are, treatment completion is low and attendance/uptake poor [24–27]. In one US study, only 17% of 658 men seeking alcohol treatment who had perpetrated IPA in the past year were referred to a perpetrator programme by the alcohol treatment service, and only 15 (13%) enrolled in the programme [27]. Amongst 286 males convicted for intimate partner violence and court-mandated to attend a community-perpetrator programme in Spain, a significantly higher rate of intervention drop-out was reported amongst men with alcohol problems (36% vs. 23%, *p* < .05) [26].

Few perpetrator interventions have been trialled amongst men who use substances [28]. A recent review of interventions to reduce IPA perpetration by men who use substances identified only nine trials, three of which were conducted in substance use treatment services and two of these amongst men who had been arrested for IPA or court-mandated to receive the intervention [28]. Initial reviews of IPA interventions conducted concurrently with alcohol treatment [29] or integrated interventions that address both IPA and substance use [28] show some promise but were not superior to treatment as usual [28]. Some interventions to address IPA in men who use substances present a simplistic model of inebriated physical violence and struggle to integrate issues of gender, non-physical abuse and to fully consider the impact of a substance use lifestyle. Recent research has identified that it is not one factor that explains causal pathways into IPA, but rather a multilevel and multifactor explanation is required [17, 18, 30]. A nested ecological model identifies factors at 4 levels, including structural factors such as patriarchy, sub-cultural factors, for example, high tolerance for general alcohol use, familial factors, including modelling from family of origin and social learning theory, and individual factors such as high anger or high impulsivity [31]. This multifactorial response requires a more nuanced understanding of the roles of substance use in IPA perpetration that is wider than intoxicated abuse. Withdrawal and craving present as many risks as intoxication and intensify coercive control [18]. Many perpetrators report psychological problems and explain IPA as a shared response to anxiety or depression and anger, often from emotional insecurities shaped by negative childhood experiences, and mediated by substances [17].

## Rationale

IPA is not routinely identified or addressed in substance use treatment, resulting in a large proportion of perpetrators who might benefit from treatment being missed. Ultimately, training substance use treatment staff to deliver integrated interventions to address IPA in substance use treatment services would increase their reach.

We are not aware of any UK-based randomised control trials of the effectiveness of perpetrator programmes for men who use substances. Further research is needed to develop and test integrated interventions that address IPA and substance use concurrently. The provision of strategies to holistically address both may be more effective [32].

## Methods

The protocol (Version 5, 13 November 2018) complies with the guidelines of the Standard Protocol Items: Recommendations for Interventional Trials (SPIRIT) [33] (Table 1).

**Table 1:** SPIRIT Checklist

Section/item	Item no.	Description	Addressed on page number
Administrative information			
Title	1	Descriptive title identifying the study design, population, interventions, and, if applicable, trial acronym	Title page
Trial registration	2a	Trial identifier and registry name. If not yet registered, name of the intended registry	Abstract, 2
Protocol version	3	Date and version identifier	3
Funding	4	Sources and types of financial, material, and other support	15
Roles and responsibilities	5a	Names, affiliations, and roles of protocol contributors	15
	5b	Name and contact information for the trial sponsor	15
	5c	Role of study sponsor and funders, if any, in study design; collection, management, analysis, and interpretation of data; writing of the report; and the decision to submit the report for publication, including whether they will have ultimate authority over any of these activities	15
	5d	Composition, roles, and responsibilities of the coordinating centre, steering committee, endpoint adjudication committee, data management team, and other individuals or groups overseeing the trial, if applicable (see Item 21a for data monitoring committee)	14
Introduction			
Background and rationale	6a	Description of research question and justification for undertaking the trial, including summary of relevant studies (published and unpublished) examining benefits and harms for each intervention	2–3
	6b	Explanation for choice of comparators	10
Objectives	7	Specific objectives or hypotheses	3, 10–11
Trial design	8	Description of trial design including type of trial (e.g. parallel group, crossover, factorial, single group), allocation ratio, and framework (e.g. superiority, equivalence, noninferiority, exploratory)	3
Methods: participants, interventions, and outcomes			
Study setting	9	Description of study settings (e.g. community clinic, academic hospital) and list of countries where data will be collected. Reference to where list of study sites can be obtained	3
Eligibility criteria	10	Inclusion and exclusion criteria for participants. If applicable, eligibility criteria for study centres and individuals who will perform the interventions (e.g. surgeons, psychotherapists)	3, 6
Interventions	11a	Interventions for each group with sufficient detail to allow replication, including how and when they will be administered	9–10
	11b	Criteria for discontinuing or modifying allocated interventions for a given trial participant (e.g. drug dose change in response to harms, participant request, or improving/worsening disease)	13
	11c	Strategies to improve adherence to intervention protocols, and any procedures for monitoring adherence (e.g. drug tablet return, laboratory tests)	10
	11d	Relevant concomitant care and interventions that are permitted or prohibited during the trial	10
Outcomes	12	Primary, secondary, and other outcomes, including the specific measurement variable (e.g. systolic blood pressure), analysis metric (e.g. change from baseline, final value, time to event), method of aggregation (e.g. median, proportion), and time point for each outcome. Explanation of the clinical relevance of chosen efficacy and harm outcomes is strongly recommended	11–13
Participant timeline	13	Time schedule of enrolment, interventions (including any run-ins and washouts), assessments, and visits for participants. A schematic diagram is highly recommended (see figure)	Fig. 1, Table 2, 6–8
Sample size	14	Estimated number of participants needed to achieve study objectives and how it was determined, including clinical and statistical assumptions supporting any sample size calculations	13–14
Recruitment	15	Strategies for achieving adequate participant enrolment to reach target sample size	10
Methods: assignment of interventions (for controlled trials)			
Allocation:			
Sequence generation	16a	Method of generating the allocation sequence (e.g. computer-generated random numbers), and list of any factors for stratification. To reduce predictability of a random sequence, details of any planned restriction (e.g. blocking) should be provided in a separate document that is unavailable to those who enrol participants or assign interventions	8
Allocation concealment mechanism	16b	Mechanism of implementing the allocation sequence (e.g. central telephone; sequentially numbered, opaque, sealed envelopes), describing any steps to conceal the sequence until interventions are assigned	8
Implementation	16c	Who will generate the allocation sequence, who will enrol participants, and who will assign participants to interventions	8
Blinding (masking)	17a	Who will be blinded after assignment to interventions (e.g. trial participants, care providers, outcome assessors, data analysts), and how	8
	17b	If blinded, circumstances under which unblinding is permissible, and procedure for revealing a participant’s allocated intervention during the trial	8
Methods: data collection, management, and analysis			
Data collection methods	18a	Plans for assessment and collection of outcome, baseline, and other trial data, including any related processes to promote data quality (e.g. duplicate measurements, training of assessors) and a description of study instruments (e.g. questionnaires, laboratory tests) along with their reliability and validity, if known. Reference to where data collection forms can be found, if not in the protocol	11–13
	18b	Plans to promote participant retention and complete follow-up, including a list of any outcome data to be collected for participants who discontinue or deviate from intervention protocols	10
Data management	19	Plans for data entry, coding, security, and storage, including any related processes to promote data quality (e.g. double data entry; range checks for data values). Reference to where details of data management procedures can be found, if not in the protocol	11–13
Statistical methods	20a	Statistical methods for analysing primary and secondary outcomes. Reference to where other details of the statistical analysis plan can be found, if not in the protocol	14
	20b	Methods for any additional analyses (e.g. subgroup and adjusted analyses)	14
	20c	Definition of analysis population relating to protocol non-adherence (e.g. as randomised analysis), and any statistical methods to handle missing data (e.g. multiple imputation)	14
Methods: monitoring			
Data monitoring	21a	Composition of data monitoring committee (DMC); summary of its role and reporting structure; statement of whether it is independent from the sponsor and competing interests; and reference to where further details about its charter can be found, if not in the protocol. Alternatively, an explanation of why a DMC is not needed	13–14
	21b	Description of any interim analyses and stopping guidelines, including who will have access to these interim results and make the final decision to terminate the trial	13
Harms	22	Plans for collecting, assessing, reporting, and managing solicited and spontaneously reported adverse events and other unintended effects of trial interventions or trial conduct	13
Auditing	23	Frequency and procedures for auditing trial conduct, if any, and whether the process will be independent from investigators and the sponsor	13
Ethics and dissemination			
Research ethics approval	24	Plans for seeking research ethics committee/institutional review board (REC/IRB) approval	15
Protocol amendments	25	Plans for communicating important protocol modifications (e.g. changes to eligibility criteria, outcomes, analyses) to relevant parties (e.g. investigators, REC/IRBs, trial participants, trial registries, journals, regulators)	15
Consent or assent	26a	Who will obtain informed consent or assent from potential trial participants or authorised surrogates, and how (see Item 32)	6-8
Confidentiality	27	How personal information about potential and enrolled participants will be collected, shared, and maintained in order to protect confidentiality before, during, and after the trial	14
Declaration of interests	28	Financial and other competing interests for principal investigators for the overall trial and each study site	15

### Aim

This trial will assess the feasibility of conducting an evaluation trial of an integrated intervention to address both substance use and IPA perpetration to men in substance use treatment.

### Design

A multicentre, parallel group, individually randomised controlled feasibility trial with a nested formative evaluation, comparing an integrated intervention to reduce IPA + usual substance use treatment, to usual substance use treatment only for men in substance use treatment.

### Setting and participants

One hundred and eight male participants will be recruited from NHS and voluntary organisation community substance use treatment services in three regions in England (two services in London, two services in the West Midlands and two services in the South West). Sets of up to 18 men per treatment service will be randomised. Three sets of the 16-week ADVANCE intervention, one in each region, will be completed (cycle 1) and a formative evaluation undertaken to inform the implementation of cycle 2 (a further three sets of the intervention, one in each region). Up to 18 men per set will randomised per cycle.

The female current or former partners of men recruited to the trial will be offered support from an integrated safety service and invited to provide outcome data for the trial. Based on recruitment figures from other evaluations of perpetrator programs [34] and a recent UK-based perpetrator intervention [35], it is estimated that up to 70% of their current or former female partners will be recruited (*n* = 76).

### Inclusion and exclusion criteria

Men attending the substance use treatment services are eligible if:

1. They have perpetrated IPA towards a current or ex female partner in the last 12 months
2. Have had face-to-face/phone/text/social media contact at least once with that partner in the last 12 months
3. Plan to stay in the current location for the next 6 months
4. Agree to provide contact details of current and/or ex female partner
5. Able to understand and communicate in English

Men will be excluded if any of the following apply:

1. Current restraining orders prohibiting them or anyone on their behalf (e.g. the women’s support worker or the researcher) from contacting their current or ex female partner
2. Pending court cases for IPA as it is uncertain how they will be sentenced (i.e. they may not be able to participate in the trial)
3. Pending child protection hearings to ensure that participating in the trial could not be used to influence proceedings
4. Attending an intervention for IPA perpetration

For eligible men following the screening, the keyworker at the substance use treatment service will assess men’s suitability to participate in the trial.

For women whose current or former partners are participating in the trial, inclusion criteria include

1. Being aged 18 years or older
2. An ability to understand and communicate in English

Women will be excluded if any of the following apply:

1. Pending court cases for IPA
2. Pending child protection hearings

In exceptional circumstances, clinicians can override the inclusion criteria to ensure that female current or former partners are safeguarded including (1) where the female and male participant share a mobile phone and (2) where the female partner lives outside the UK and therefore integrated safety support cannot be provided—neither will be eligible to take part in the feasibility trial.

Male current or former partners and non-English speaking female current or former partners of men in the trial will not be invited to provide outcome data but will be offered support for their IPA victimisation.

### Recruitment

**Fig. 1:**
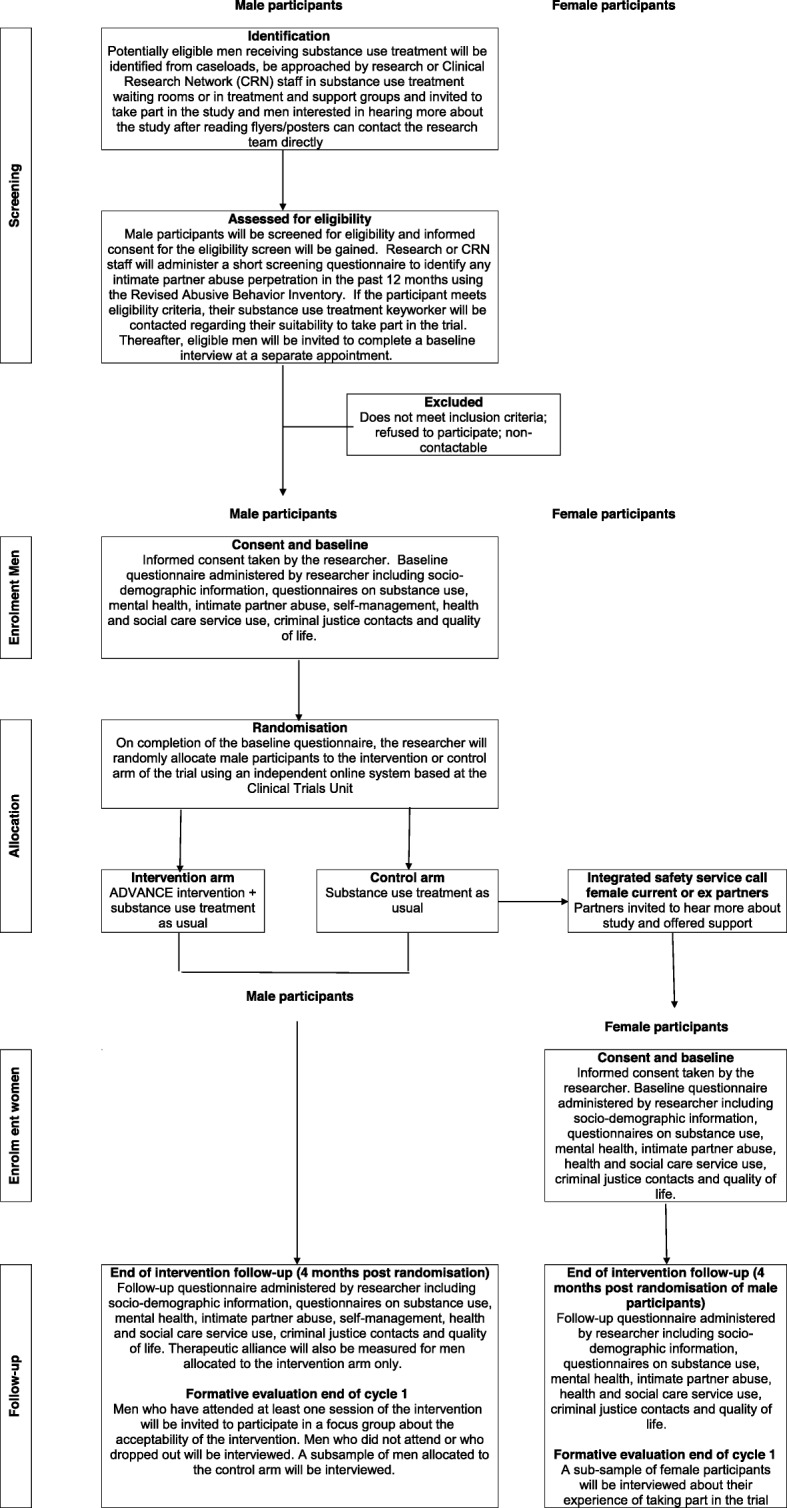
Flow of participants through the trial

Participation in the trial requires a two-stage informed consent process. Firstly, men must consent to be screened for eligibility. If eligible after screening, men then consent to take part in the trial. Researchers will screen potential male participants for eligibility using the Revised Abusive Behavior Inventory [36]. Men already attending substance use treatment will be identified in substance use treatment waiting rooms, via keyworkers or will contact researchers from details on flyers/posters advertising the research (Fig. 1). Researchers will also attend support groups delivered in the substance use service to explain the study and invite interested men to discuss the study further. Men who report this is their first appointment at the service will not be screened as they may not be enrolled in the service following assessment. To be eligible to participate in the trial, male participants have to be receiving treatment from the participating substance use service.

Researchers will explain the study to interested men, provide them with an information sheet and gain informed consent to undertake the screening, including limitations to confidentiality and consent to discuss the screening and their suitability to take part in the trial with their keyworker. Consent forms will be counter-signed in triplicate by both the male participant and the researcher. Male participants will receive a copy of the signed consent form; a copy will be filed in the Investigator Site File and a copy will be attached to the letter to the male participant’s keyworker at the substance use treatment service. This letter will inform the keyworker that their client has screened positive for past year IPA and identify areas of risk (e.g. threat/use of a weapon against partner or choking/strangulation of partner) for potential further assessment by the keyworker. It will also include the name of their client’s current and/or former female partner. Keyworkers then are able to consider the suitability of their client to participate in the trial (e.g. cognitive deficit or mental health problems that may limit their ability to participate).

If eligible, following screening and their keyworkers deem the participant suitable to take part, male participants will complete a baseline assessment administered by the researcher (see Section 3.14). The researcher will again explain the study to eligible men, provide them with an information sheet and get their informed consent to participate in the trial prior to administering the baseline questionnaire. In addition, men will complete a consent quiz [37] to ensure they understand the trial procedures. Researchers will clarify any incorrect responses with the participant. Men will be randomised to treatment conditions following baseline assessment. Their keyworkers will be informed of which group they have been allocated to by a letter from the researcher. All interviews with men will be conducted in a private room of the substance use treatment service.

Following baseline assessment and randomisation of male participants, their current or former female partners will be contacted by telephone by an integrated safety service worker to (1) inform them their current or former partner is participating in the trial; (2) to offer them support for IPA victimisation; and (3) invite them to participate in the research by providing outcome data. Women can accept the offer of support without participating in the research and vice-versa. If women are interested in participating in the research, their contact details will be passed to the research team, who will contact them within 1 week and arrange a time for an initial interview to be conducted with the researcher (see Section 3.14). At this meeting, the researcher will explain the trial to the woman and gain informed consent prior to administering the baseline interview. Women will be interviewed in a women’s support service, a substance use treatment service, a children’s centre, or in exceptional cases, it may be possible for two researchers to interview the women in her home.

After each interview, male and female participants will be given contacts for local and national helplines and services. If specific risk issues are identified within the baseline assessment, appropriate steps will be taken to manage this risk, including disclosure to the participant’s keyworker (for male participants), and/or to the integrated safety worker (for women participants).

### Follow-up

Male and female participants will be followed-up at the end of the intervention (approximately 4 months post-randomisation) (Table 2). Reminder telephone calls or texts will be sent in advance to arrange and confirm a time for follow-up interview.

**Table 2:** Schedule of enrolment, interventions, and assessments

Study phase				
Timepoint	Screening	Baseline and randomisation	Intervention delivery	Follow-up
Enrolment:				
Informed consent	X	X		
Eligibility screen	X			
Baseline interview		X		
Allocation		X		
Follow-up interview				X
Interventions:			(16 weeks)	
Intervention + TAU				
TAU				
Assessments:				
Childhood		X		
Substance use	X (males only)	X		X
Mental health		X		X
Intimate partner abuse	X (males only)	X		X
Quality of life		X		X
Service use		X		X
Desirable responding		X (males only)		X (males only)
Motivation to change behaviour		X (males only)		
Self-management		X (males only)		X (males only)
Therapeutic alliance				X (males in intervention arm only)

### Method of randomisation

Male participants will be allocated to the intervention group+ substance use treatment as usual (TAU) or TAU only (ratio 1:1) by site via an independent online system based at the Clinical Trials Unit. Allocation will be at the level of the individual participant, using randomly varying block sizes, stratified by a combination of sites and cycles (18 participants per site/cycle stratum).

### Blinding

The statisticians will be blind to the trial condition throughout the feasibility trial. The health economists will not be blind to the allocation as the intervention costs will have to be assigned to the correct treatment arm. Researchers will not be blind to the group allocation of participants, as they will also be responsible for participant recruitment and reminder telephone calls to attend intervention sessions.

### Intervention arm

**Fig. 2:**
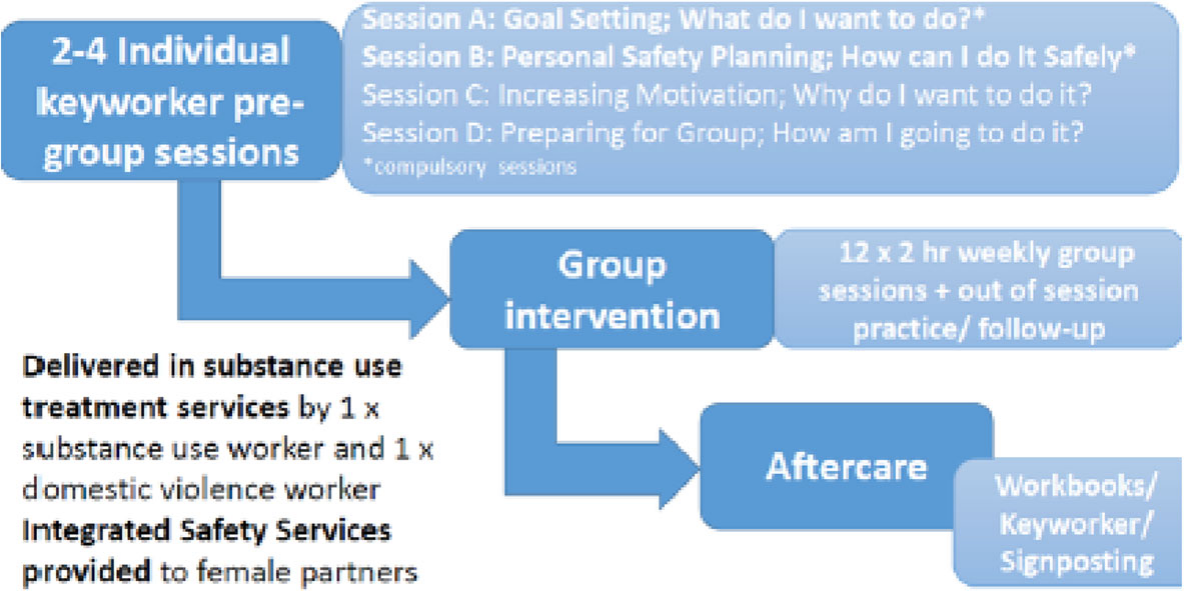
ADVANCE intervention

The TIDieR (Template for Intervention Description and Replication) Checklist was used to describe the intervention [38] (Additional file 1: Table S3). The Behaviour Change Wheel, incorporating the COM-B model (capability, opportunity, and motivation for behavioural interventions) was used to provide a framework to develop the ADVANCE intervention [39]. The ADVANCE intervention is voluntary, i.e. men are not mandated to attend by the court. Within the COM-B model, the intervention thus considered how capability, opportunity, and motivation for each could be addressed and which type of intervention would be best to enact each. Thus, modelling, enablement, education, incentivisation, and training are included in the intervention. In the ADVANCE intervention, capability is enhanced by increasing participants’ knowledge and skills based on the self-regulation model. In particular, awareness around crisis planning and self-management is raised and automatic thoughts and beliefs are challenged by evaluating the consequences. Opportunity is provided to model and promote positive behaviour within intervention sessions, and out-of-session tasks to generalise learning. Each week, sessions were reviewed to reinforce positive achievements. Finally, goal planning is used to motivate participants and reinforcement of motivation is achieved through ongoing personal support and incentives. The ADVANCE intervention is manualised comprising 2–4 individual sessions (2 need to be completed before beginning the group intervention) with a keyworker to set goals, develop a personal safety plan, and increase motivation and readiness followed by a 12-week group intervention (Fig. 2) (Gilchrist E, Johnson A, Stephens-Lewis D, Kirkpatrick S, McMurran M, Gilchrist G: Designing an implementation intervention with the Behaviour Change Wheel for Men in Substance Use Services who have been abusive to an intimate female partner, in preparation). Given the high level of trauma, family disruption, abuse, and neglect in this population, interventions targeting IPA in men who use substances require a trauma-informed approach [40]. Recent approaches with forensic populations indicate that strengths-based approaches, designed for a range of learning styles, avoiding shame and judgement are more effective [41, 42]. Three potential targets for effective IPA change were identified as reducing pro-abuse attitudes including locating causality for abuse in substance use, managing distress individually and pro-socially, and learning effective, non-abusive behavioural responses within relationship negotiations. The ADVANCE intervention focuses on developing participants’ strengths and developing healthy, non-abusing relationships. Two main models to enable change were selected (1) personal goal setting, to work with individual goals, and to build genuine motivation through alignment with personal values and facilitate change by breaking larger longer-term aims into specific small steps that can be more easily achieved, reinforcing motivation through these small achievements and (2) informed by the self-regulation model which suggests that building self-management in one domain enables more effective self-management across other domains. The intervention requires individuals to set specific goals for reducing risk, such as changing substance use, and for developing a prosocial lifestyle in terms of work, leisure, health, and accommodation. Underpinning the goal-focused approach is the need to improve self-regulation of behaviours, achieved by identifying and changing cues, appraisals (thoughts), emotions, behaviours, and consequences. Throughout the ADVANCE intervention, behaviour change skills are introduced and practised. The 12 group sessions will address the following:

- Session 1. Introduction to the group
- Session 2. Managing myself
- Session 3. Behaviour analysis + gender
- Session 4. Impact of intimate partner abuse
- Session 5. Children, parenting, substance use + intimate partner abuse
- Session 6. Relating
- Session 7. Improving communication
- Session 8. Dealing with distress
- Session 9. Planning to be better
- Session 10. Positive relationships
- Session 11. New futures, peoples plans + positive activities
- Session 12. Recap what we have learned

Pre-group individual sessions will be delivered by keyworkers in substance use treatment service. The group intervention will also be delivered in substance use treatment services by two trained facilitators, where possible one female and one male. Keyworkers or facilitators will check-in by phone with participants between group sessions to address any issues arising during the session. Participants will be given out of session practice exercises. Facilitators will send a weekly feedback to participants’ keyworkers by email after each session to update them on progress, safeguarding issues and risk. Group sessions will be video recorded with participants’ consent and checked for fidelity. Only those consenting to being video recorded would be able to take part in the trial.

Fortnightly, integrity support meetings will be held with facilitators to discuss delivery issues and address any expected problems for delivery of the next two sessions. Four case management meetings will take place with facilitators (and keyworkers where possible) and the integrated safety service workers over the course of the intervention to consider risk. Aftercare is provided in the form of workbooks for men, keyworker guidance, and signposting.

Contingency management, recommended by the National Institute for Health and Care Excellence [43], will be used to encourage motivation and retention of participants in the group intervention by offering incentives to attend the ADVANCE integrated intervention. Participants in the intervention arm only will receive a £5 voucher (for a shop or service of their choosing) for attending each of the 12 sessions up to a total of £60. These will be awarded at session 6 and session 12 of the group intervention. If a participant attends all 12 sessions an additional £10 ‘bonus’ voucher will be available. Travel will also be reimbursed, and refreshments provided.

The integrated safety service will also contact the female current or former partners of males in the intervention arm on at least three occasions to update them on their current or ex-partner’s overall progress within the intervention. They will also contact women if there is anything that they need to know to keep themselves and their families safe.

### Control arm

Men in both treatment arms will receive substance use TAU including group work, individual sessions, mutual aid, and opiate substitution treatment. Integrated safety services and individualised support are offered to all participants’ female current or ex-partners as needed, regardless of the allocation to a group of their male partners.

Data from naturalistic studies found that the relative risk for IPA after substance use treatment was 2–3 times greater amongst those who relapsed compared to those who successfully completed treatment [44], potentially as a result of a reduction in alcohol use and improved relationship functioning. Therefore, the selection of substance use TAU as a comparator for this feasibility study is justified. Moreover, men’s keyworkers will be informed that their client has perpetrated IPA in the past 12 months regardless of group allocation and may choose to address this as part of TAU.

### Fidelity

All group intervention sessions will be video recorded with participants’ and facilitators’ consent. Facilitators’ fidelity of the delivery of the intervention manual will be assessed by a trained observer using a pre-defined checklist for each session of the intervention. Video recordings will allow the same observer to assess treatment integrity, the degree to which treatment is implemented as intended across sites. Sessions are videotaped for assessment of treatment fidelity, therapist skill, and competency in delivering the interventions and the discriminability of the study treatments. Treatment fidelity will be assessed using an adapted form of the Yale School of Medicine Adherence and Competence Scale [37], shown to be successful at discriminating key therapy content and skill domains [38]. A series of checklists are created to tap into key unique components of specific therapies (e.g. experimental condition vs. control) as well as a series of scales tapping common factors (e.g. assessment of substance use and general functioning).

### Formative evaluation

At the end of each group session, men will be asked to self-complete a brief evaluation form. Facilitators will complete a brief treatment adherence form after each session. All TAU contacts will be recorded by keyworkers for men (regardless of treatment arm). Integrated safety support workers will record contacts with the current or ex female partners of men in both treatment arms. Focus groups with staff at the end of each cycle of the intervention will examine their experiences of delivering the intervention. After completion of the first cycle of the intervention at each site, interviews with participants will explore their experiences and acceptability of attending the intervention and participating in the trial.

### Feasibility measures

The primary objective of this study is to demonstrate the feasibility of conducting a trial to evaluate the intervention, including the feasibility and acceptability of delivering the intervention in substance use treatment and the feasibility of outcome measure collection on perpetrators and their current or former female partners. The following feasibility parameters will be examined: rates of consent, recruitment, and follow-up by site and group allocation. The estimation of background variability will inform sample size calculations for a future evaluation trial.

Men’s TAU attendance for both treatment arms, attendance, and retention in the ADVANCE intervention for men allocated to the treatment arm, and numbers of current or former female partners accepting the offer of support from the integrated safety service will also be collected.

### Participant-centred outcome measures

The suitability and acceptability of outcome measures will be assessed to determine the feasibility of including them in a future effectiveness trial. Researchers’ perceptions of participants’ understanding (e.g. of language and meaning of questions) and acceptability (e.g. if participant refuses to answer or gets annoyed/frustrated or asks to end the interview) will be recorded for each outcome measure using a pre-determined rating scale (1—lowest rating to 3—highest rating for understanding and acceptability). The following outcome measures will be assessed at baseline for men and initial interview for women and at the end of the intervention (4 months post-randomisation of men) for men in both treatment arms and their current or former female partners (unless otherwise specified) (Table 2).

### Substance use

*The Alcohol Use Disorders Identification Test* (AUDIT) (included in the baseline questionnaire only) is a 10-item screening tool used to assess alcohol use in the past 12 months [45]. Scores range from 0 to 40. Total scores of 8 or more for men (7 or more for women) indicate hazardous and harmful alcohol use, and scores of 20 or above indicate alcohol dependence.

*The Drug Use Disorders Identification Test* (DUDIT) (included in the baseline questionnaire only) is an 11-item screening tool used to *identify drug*-related problems [46]. Scores range from 0 to 44. Total scores of 6 or more for men (2 or more for women) indicate drug-related problems, and scores of 25 or more indicate drug dependence.

As the AUDIT and DUDIT assess substance use in the past 12 months they will not be used to measure substance use outcomes post-intervention (4 months post-randomisation). Instead, the average amount on a using day and number of days substances used in each of the past 4 weeks will be recorded using the Treatment Outcome Profile [47] at baseline and follow-up. The number of days in the past 4 weeks that problems with particular substances were experienced will also be recorded using *The Addiction Severity Index* [48] as will current treatment for substance use.

### Mental health

*The Patient Health Questionnaire* (PHQ-9) is a 9-item scale measuring depressive symptoms in the past 2 weeks [49]. The total score ranges from 0 to 27. A score ≥ 10 has a sensitivity of 88% and a specificity of 88% for major depression.

*The Generalised Anxiety Disorder Assessment* (GAD-7) is a 7-item scale measuring general anxiety symptoms in the past 2 weeks [50]. The total score ranges from 0 to 21. A score of ≥ 10 represents a reasonable cut off point for identifying cases of GAD.

*The Primary Care Post-Traumatic Stress Disorder (PTSD) Screen* (PC-PTSD-5) is a 5-itccem screen for PTSD in the past month, with scores ranging from 0 to 5 [51]. A score of ≥ 3 indicates PTSD.

*The Standardised Assessment of Personality—Abbreviated Scale* (SAPAS) (included in male baseline questionnaire only) is an 8-item screening interview for personality disorder [52] enquiring about a person’s behaviour in general rather than a specific time frame. Scores range from 0 to 8. A score ≥ 3 correctly identified the presence of DSM-IV personality disorder in 90% of participants.

### Intimate partner abuse (victimisation and perpetration) in the past 4 months

*The Abusive Behaviour Inventory Revised* (ABI-R) is a 25-item reliable tool to measure experiences of physical (13 items), psychological (9 items), and sexual abuse (3 items) victimisation and perpetration [36]. Each item can be scored from 1 (never) to 5 (very frequently). The higher the score on each subscale, the greater the frequency of abuse.

Controlling behaviours in relation to both perpetration and victimisation will be assessed using four adapted questions from the 24 item *Revised Controlling Behaviours Scale* (CBS-R) [53]: smash your partner’s property when annoyed/angry; want to know where your partner went and who they spoke to when not together; tell your partner they were going mad; and try to restrict time your partner spent with family or friends. Total score for the 4 questions ranges from 0 to 16; the higher the score, the greater the frequency of a partner using controlling behaviours.

*The Communications Patterns Questionnaire-Short Form* (CPQ-SF) [54] is an 11-item self-assessment of “spouses’ perceptions of marital interactions … to indicate the representativeness of that description for the conflict and communication patterns in their relationship”: male demand/female withdraw; female demand/male withdraw; original total demand/withdraw; alternate demand/withdraw; criticise/defend and positive interaction. Higher scores on each subscale suggest which communication pattern is likely to be used during conflict interactions.

Two scales (9 items) from *The Intimate Partner Violence Responsibility Attribution Scale* (IPVRAS) (included in the male baseline and follow-up questionnaires only) will assess perpetrators’ responsibility attribution to the victim (V) and responsibility attribution to the offender’s (i.e. perpetrator’s) personal context (O) [55]. The higher the score, the more responsibility attributed to the victim (V) or offender’s personal context (O).

The 12-item anger subscale from *The Propensity for Abusiveness Scale* (PAS) (included in the male baseline and follow-up questionnaires only) is included. The PAS predicts the perpetration of physical and emotional abuse. Scores on the anger subscale range from 12 (completely undescriptive of you) to 60 (completely descriptive of you) [56].

Two questions from a non-validated scale on the use of social media in relation to perpetration and victimisation of a partner will be included [57] (e.g. ‘Used mobile technology to check her location’). Total scores range from 2 (never) to 10 (very frequently); the higher the score, the greater the frequency of abuse using social media.

Four questions will be used from a non-validated scale to assess the use of children against a partner [35] (e.g. “Asked the children to report on what your current or former partner is doing or where she has been”). Total scores range from 4 (never) to 20 (very frequently); the higher the score, the greater the frequency of using children against a partner/the greater the frequency of having a partner use children against you.

There will be a question on the frequency of stopping a partner from leaving the house against their will/being stopped from leaving the house will be scored from 1 (never) to 5 (very frequently). Two questions on stalking behaviours scoring from 2 (never) to 5 (very frequently); the higher the score, the greater the frequency of experiencing or perpetrating stalking behaviour.

### Self-management

The 13-item *Brief Self-Control Scale* (BSCS) enquires about typical dispositional self-regulatory behaviours using two factors of self-control: restraint and impulsivity [58] (included in the male baseline and follow-up questionnaires only). Total scores range from 13 (not at all like me) to 65 (very much like me).

### Quality of life

*The ICEpop CAPability measure for Adults* (ICECAP-A) is used to assess a person’s well-being using a capability approach [59]. It has five attributes: **attachment** (an ability to have love, friendship, and support); **stability** (an ability to feel settled and secure); **achievement** (an ability to achieve and progress in life); **enjoyment** (an ability to experience enjoyment and pleasure), and **autonomy** (an ability to be independent), each with four levels of capacity. Tariff values range from 1 (full capability) to 0 (no capability).

*The EQ-5D-3L* consists of a descriptive system and a Visual Analogue Scale (VAS). The descriptive system assesses five dimensions of a person’s health state (mobility, self-care, usual activities, pain/discomfort, and anxiety/depression) on the day of administering, each with three response levels: no problems, some problems, and extreme problems [60]. The tariff index score based on the descriptive profile ranges from 1 (perfect health) to – 0.594 (worst health), with death anchored at 0 for the UK valuation set [61]. The EQ VAS records the respondent’s current self-rated health on a vertical visual analogue scale from 0 (worst imaginable health) to 100 (best imaginable health).

### Healthcare, social, and other legal and civil services use and criminal justice contacts

Participants’ utilisation of primary and secondary healthcare, social services, and other legal and civil services, including the prescribing of medication, and their contacts with criminal justice will be recorded retrospectively by self-report questionnaire.

### Experience of the group intervention (males allocated to the intervention arm, follow-up questionnaire only)

The 12-item *Working Alliance Inventory–Short Revised (WAI-SR) client version* measures three key aspects of therapeutic alliance: agreement on the tasks of therapy, agreement on therapy goals, and affective bond development [62]. *Scores range from 5 to 60,* with higher scores indicating a better therapeutic alliance between client and therapist. The patient version of the *California Psychotherapy Alliance Scale- Short Form* [63] has 12 items that measures the alliance in psychotherapy across 4 subscales: the patient working capacity, patient commitment, working strategy consensus, and therapist understanding and involvement. The total score is the mean of these four subscales.

The following will also be assessed and may be used as explanatory variables.

### Socio-demographic information

Data on participants’ relationship status, age, gender, ethnicity, highest education level attained, living arrangements, current employment status, number of children, and their living arrangements will be recorded.

### Childhood

The occurrence of ten *Adverse Childhood Experiences* (ACE) (e.g. physical, sexual, emotional abuse, neglect, household dysfunction) experienced before the age of 18 were assessed to produce an ACE score of cumulative childhood stress [64] (assessed in baseline questionnaire only). Scores range from 0 to 10; the higher the score the greater the childhood adversity experienced.

### Desirable responding

The *Balanced Inventory of Desirable Responding Short Form* (BIDR-SF) [65] (administered to male participants only). The BIDR-SF consists of two subscales: Self-Deceptive Enhancement (honest but overly positive responding) and Impression Management (bias toward pleasing others or ‘deliberate self-presentation’). Higher scores indicating more desirable responses. IPA scores will be adjusted for BIDR-SF scores.

### Motivation to change behaviour

*The URICA-Domestic Violence* (URICA-DV), administered to male participants only, is a 32-item measuring perpetrators’ readiness to end their violence [66]. URICA-DV includes four subscales to measure stages of change pre-contemplation, contemplation, action, and relapse. Responses are given on a 5-point Likert scale ranging from 1 (strong disagreement) to 5 (strong agreement).

#### Database systems

A web based electronic Case Report Forms system (InferMed MACRO) will be used to collect baseline and outcome data. The system is Good Clinical Practice compliant with full audit trail and database lock functionality and a range of validations will be programmed to minimise data entry errors. No data analysis will be undertaken until databases are locked.

Ten percent of coded questionnaires and 10% of entered data by site will be verified by the trial manager to check for errors. If coded questionnaires and data entered are found to contain errors, a further 10% will be checked.

#### Ethical issues

The trial will be conducted in compliance with the principles of the Declaration of Helsinki [67], the principles of Good Clinical Practice, and all of the applicable regulatory requirements (UK data protection laws (meaning the Data Protection Act 1998 until 24 May 2018, and from 25 May 2018 the European Union General Data Protection Regulation (GDPR) and applicable UK legislation that enshrines GDPR into UK law).

As part of the informed consent process, participants will be advised and provided guidance about confidentiality and the limits to it. Significant risk of future harm to self or others will be disclosed to their keyworker or the duty worker in the substance use service or the integrated safety service where the interview is taking place, or to the relevant authorities.

The trial may be prematurely discontinued by the Sponsor or Chief Investigator on the basis of new safety information or for other reasons given by the Data Monitoring and Ethics Committee/Trial Steering Committee, regulatory authority or ethics committee concerned. The trial may also be prematurely discontinued due to lack of recruitment or upon advice from the Trial Steering Committee. If the study is prematurely discontinued, active participants will be informed and no further participant data will be collected.

All serious adverse events resulting from participation in the trial will be reported to the Ethics Committee within 48 h of receiving the report.

#### Research reimbursement

Male and female participants will be reimbursed in cash or vouchers for their time for completing baseline or follow-up interviews (£10) and taking part in focus groups or qualitative interviews (£20). All research participants will receive £5 travel expenses for attending a research interview.

#### Sample size calculation

The sample size for a feasibility study should allow parameters required to inform the design of the definitive randomised controlled trial to be estimated. To estimate a standard deviation for a sample size calculation of a future evaluation trial, a sample of at least 50 participants is recommended [68]. Therefore, to recruit a sample size of 60 female current or former partners (i.e. 30 whose current or ex male partners in the intervention arm and 30 whose partners are in the control arm), and taking a conservative retention rate of 80% of female current or former partners at end of intervention assessment (4 months post-randomisation), we will recruit 76 female current or former partners. It is estimated that around 70% of current or former female partners will agree to take part in the trial, therefore approximately 108 male participants will need to be recruited to obtain a sample of 76 female current or former partners.

#### Statistical analysis

This feasibility trial will estimate parameters needed for planning an efficacy trial including estimating various rates (e.g. recruitment, randomisation, follow-rates) and estimating the within-trial arm standard deviation, the pre-post correlation, and the Intracluster Correlation Coefficient (*ICC*) for possible outcome measures to inform the sample size calculation of a future randomised control trial. Outcome measures will be rated by the researcher for each participant for their suitability and acceptability and the mean scores summarised for each outcome. Outcome measures will be described by trial arm. Intervention effect sizes (e.g. incidence rate ratios for the count outcome and mean differences for continuous outcome measures) will be estimated for various outcome measures, but no formal hypotheses testing will be carried out.

#### Economic analysis

The costs of providing the intervention will be collected from local data sources to estimate the incremental cost of delivering the ADVANCE intervention over and above substance use TAU. Questionnaires on the use of primary and secondary health and social care services and contacts with criminal justice services by men (and their female current or ex-partners) in the intervention and control groups will record quantities of resource use. This resource use will be multiplied by unit costs to estimate a cost profile for each participant. The returned questionnaires will be analysed to assess the acceptability of data collection methods, identify barriers to data collection and highlight the most commonly used services in this population. A more appropriate questionnaire for an efficacy trial can be developed based on the feasibility trial data. A cost profile will be estimated for each participant, including trial-related costs and wider service use costs and presented by treatment arm.

#### Qualitative analysis

Focus groups and semi-structured interviews will be digitally recorded and transcribed verbatim. Data will be organised and coded using NVivo. Multiple coders will enhance the rigour of the analysis. Framework analysis will be used for qualitative data analysis using five steps: familiarization; identifying a thematic framework; indexing; charting; and mapping and interpretation [69]. Collecting multiple perspectives data (e.g. from male and female participants, keyworkers, intervention facilitators, and women’s support staff) in a qualitative formative evaluation will provide a better understanding of the intervention’s implementation. The *framework* approach allows the exploration of patterns in themes across different participants and groups of participants.

#### Data management

In accordance with the Trial Terms of Reference, the Data Monitoring and Ethics Committee will periodically review overall safety data to determine patterns and trends of events, or to identify safety issues, which would not be apparent on an individual case basis and report their findings to the Trial Steering Committee who will provide overall supervision of the feasibility trial.

#### Data handling and storage

Participants’ contact details will be stored on a secure password protected server for 3–6 months after the study has ended. Consent forms will be stored at each site in a locked cabinet and will not contain the participant identification number. Paper copies of questionnaires will be stored separately from the consent forms and contain only a participant identification number. A unique identification code will be assigned to electronic sound files and transcripts of the focus groups, known only to appropriate members of the research team. Any personal information required will also be coded with this identification number and kept in a password protected server. Audio files will not contain participant’s names and will not be stored on the recording device. Audio files will be downloaded onto a secure server. As soon as the audio file is checked on the server, it will be deleted from the recording device. Any quotes published will be anonymous further protecting participant confidentiality. Recordings will be archived in a secure location for a minimum period of 7 years.

## Discussion

IPA remains a leading contributor to disease burden [70]. It is estimated that the annual cost of domestic abuse in England and Wales is over £66 billion, mainly due to the cost of physical and emotional harms experienced by victims/survivors (£47 billion) [71]. There is a need to find effective interventions to reduce IPA perpetration, especially amongst high-risk groups such as substance users. Current provision of community perpetrator groups does not meet demand nor is it tailored to the specific needs of men in substance use treatment. This unmet need led to the development of the ADVANCE intervention. It is hoped that delivering tailored IPA interventions in substance use treatment will increase their ability to reach men who may not otherwise be referred to perpetrator interventions and result in better outcomes for male perpetrators and improved wellbeing for their female partners.

This feasibility trial, and nested formative evaluation, will inform the design of a future trial to evaluate the clinical efficacy and cost-effectiveness trial.

### Trial status

The trial sponsor is South London and Maudsley NHS Foundation Trust. The screening began on 5 July 2018, with the first male participant randomised on 17 July 2018. Recruitment will end on 7 May 2019.

## Supplementary information

**Additional file 1: Table S3.** The TIDieR (Template for Intervention Description and Replication) Checklist. Information to include when describing an intervention and the location of the information.

## Data Availability

Not applicable.

## Abbreviations

ADVANCE: Advancing theory and treatment approaches for males in substance misuse treatment who perpetrate intimate partner violence
GDPR: General Data Protection Regulation
IPA: Intimate partner abuse
IPV: Intimate partner violence
ISS: Integrated safety service
RCT: Randomised controlled trial
SPIRIT: The Standard Protocol Items: Recommendations for Interventional Trials
TAU: Treatment as usual

## References

[1] Home Office Domestic Violence and Abuse. 2018. https://www.gov.uk/guidance/domestic-violence-and-abuse. Accessed 10 Apr 2019.

[2] Gutierres SE, Van Puymbroeck C (2006). Childhood and adult violence in the lives of women who misuse substances. Aggress Violent Behav.

[3] Chermack ST, Murray RL, Walton MA, Booth BA, Wryobeck J, Blow FC (2008). Partner aggression among men and women in substance use disorder treatment: correlates of psychological and physical aggression and injury. Drug Alcohol Depend.

[4] World Health Organization (2013). Global and regional estimates of violence against women: prevalence and health effects of intimate partner violence and nonpartner sexual violence.

[5] Monckton-Smith J, Williams A, Mullane F (2014). Domestic abuse, homicide and gender.

[6] Dixon L, Graham-Kevan N (2011). Understanding the nature and etiology of intimate partner violence and implications for practice and policy. Clin Psychol Rev.

[7] Dahlberg LL, Krug EG, Krug E, Dahlberg LL, Mercy JA, Zwi AB, Lozano R (2002). Violence - a global public health problem. World Report on Violence and Health.

[8] Gilchrist G, Blazquez A, Segura L, Geldschläger H, Valls E, Colom J, Torrens M (2015). Factors associated with physical or sexual intimate partner violence perpetration by men attending substance misuse treatment in Catalunya: A mixed methods study. Crim Behav Ment Health.

[9] Gilchrist G, Radcliffe P, Noto AR, d'Oliveira AF (2017). The prevalence and factors associated with ever perpetrating intimate partner violence by men receiving substance use treatment in Brazil and England: a cross-cultural comparison. Drug Alcohol Rev.

[10] Foran HM, O'Leary KD (2008). Alcohol and intimate partner violence: a meta-analytic review. Clin Psychol Rev.

[11] Leonard KE, Quigley BM (2017). Thirty years of research show alcohol to be a cause of intimate partner violence: Future research needs to identify who to treat and how to treat them. Drug Alcohol Rev.

[12] Smith PH, Homish GG, Leonard KE, Cornelius JR (2012). Intimate partner violence and specific substance use disorders: findings from the national epidemiologic survey on alcohol and related conditions. Psychol Addict Behav.

[13] Moore TM, Stuart GL, Meehan JC, Rhatigan DL, Hellmuth JC, Keen SM (2008). Drug abuse and aggression between intimate partners: a meta-analytic review. Clin Psychol Rev.

[14] Graham K, Bernards S, Wilsnack SC, Gmel G (2011). Alcohol may not cause partner violence but it seems to make it worse: a cross national comparison of the relationship between alcohol and severity of partner violence. J Interpers Violence.

[15] Kraanen FL, Vedel E, Scholing A, Emmelkamp PM (2014). Prediction of intimate partner violence by type of substance use disorder. J Subst Abus Treat.

[16] Cafferky BM, Mendez M, Anderson JR, Stith SM (2018). Substance use and intimate partner violence: A meta-analytic review. Psychol Violence.

[17] Gilchrist G, Dennis F, Radcliffe P, Henderson J, Howard LM, Gadd D (2019). The interplay between substance use and intimate partner violence perpetration: A meta-ethnography. Int J Drug Policy.

[18] Gadd D, Henderson J, Radcliffe P, Stephens-Lewis D, Johnson A, Gilchrist G (2019). The dynamics of domestic abuse and drug and alcohol dependency. Br J Crimnol.

[19] O'Farrell TJ, Murphy CM, Stephan SH, Fals-Stewart W, Murphy M (2004). Partner violence before and after couples-based alcoholism treatment for male alcoholic patients: the role of treatment involvement and abstinence. J Consult Clin Psychol.

[20] El-Bassel N, Gilbert L, Wu E, Chang M, Fontdevila J. Perpetration of intimate partner violence among men in methadone treatment programs in New York City. Am J Public Health. 2007 Jul;97(7):1230-2.Frye V, Latka MH, Wu Y, Valverde EE, Knowlton AR, Knight KR, Arnsten JH,. 10.2105/AJPH.2006.090712 PMC1913091 17538056

[21] O'leary A (2007). INSPIRE Study Team. Intimate partner violence perpetration against main female partners among HIV-positive male injection drug users. J Acquir Immune Defic Syndr.

[22] Radcliffe P, Gilchrist G (2016). “You can never work with addictions in isolation”: Addressing intimate partner violence perpetration by men in substance misuse treatment. Int J Drug Policy.

[23] Respect Domestic Violence perpetrator programmes Commissioning Guidance for Police and Crime Commissioners 2013. http://www.senedd.assembly.wales/documents/s30732/GBV%2090b%20-%20Respect.pdf. Accessed 10 Apr 2019.

[24] Klostermann KC, Fals-Stewart W (2006). Intimate partner violence and alcohol use: Exploring the role of drinking in partner violence and its implications for intervention. Aggress Violent Behav.

[25] Timko C, Valenstein H, Lin PY, Moos RH, Stuart GL, Cronkite RC (2012). Addressing substance abuse and violence in substance use disorder treatment and batterer intervention programs. Subst Abuse Treat Prev Policy.

[26] Lila M, Gracia E, Miñana C (2017). More Likely to Dropout, but What if They Don’t? Partner Violence Offenders With Alcohol Abuse Problems Completing Batterer Intervention Programs. J Interpers Violence.

[27] Schumacher JA, Fals-Stewart W, Leonard KE (2003). Domestic violence treatment referrals for men seeking alcohol treatment. J Subst Abus Treat.

[28] Stephens-Lewis D, Johnson A, Huntley A, McMurran M, Gilchrist E, Henderson J, Howard L, Feder G, Gilchrist G. Interventions to Reduce Intimate Partner Violence Perpetration by Men Who Use Substances: A Systematic Review and Meta-Analysis of Efficacy. Trauma Violence Abuse. 10.1177/1524838019882357. 10.1177/1524838019882357 PMC8649458 31711372

[29] Tarzia L, Forsdike K, Feder G, Hegarty K. Interventions in health settings for male perpetrators or victims of intimate partner violence. Trauma Violence Abuse. 2017. 10.1177/1524838017744772. 10.1177/1524838017744772 29333972

[30] Radcliffe P, Gadd D, Henderson J, Love B, Stephens-Lewis D, Johnson A, Gilchrist G. What role does substance use play in intimate partner violence? A narrative analysis of in-depth interviews with men in substance use treatment and their current or former female partner. J Interpers Violence. 2019. 10.1177/0886260519879259. 10.1177/0886260519879259 PMC8581707 31578902

[31] Dutton DG (2006). Rethinking domestic violence.

[32] McMurran M (2012). Individual-level interventions for alcohol-related violence: A rapid evidence assessment. Crim Behav Ment Health.

[33] Chan A-W, Tetzlaff JM, Altman DG, Laupacis A, Gøtzsche PC, Krleža-Jerić K (2013). SPIRIT 2013 Statement: Defining standard protocol items for clinical trials. Ann Intern Med.

[34] Gondolf EW (2009). Outcomes from referring batterer program participants to mental health treatment. J Family Viol.

[35] Kelly L, Westmarland N. Domestic violence perpetrator programmes: steps towards change. Project Mirabal final report. London and Durham: London Metropolitan University and Durham University. 2015. https://www.dur.ac.uk/resources/criva/ProjectMirabelFinalReport.pdf. Accessed 10 Apr 2019.

[36] Postmus JL, Stylianou AM, McMahon S (2016). The Abusive Behavior Inventory–Revised. J Interpers Violence.

[37] Crane CA, Hawes SW, Mandel D, Easton CJ (2013). Informed consent: an ethical issue in conducting research with male partner violent offenders. Ethics Behav.

[38] Hoffmann TC, Glasziou PP, Boutron I, Milne R, Perera R, Moher D (2014). Better reporting of interventions: template for intervention description and replication (TIDieR) checklist and guide. BMJ.

[39] Michie S, van Stralen MM, West R (2011). The behaviour change wheel: A new method for characterising and designing behaviour change interventions. Implement Sci.

[40] Voith LA, Logan-Greene P, Strodthoff T, Bender AE (2018). A Paradigm shift in batterer intervention programming: a need to address unresolved trauma. Trauma Violence Abuse.

[41] Marshall WL, Marshall LE, Serran G, Fernandez YM (2006). Treating Sexual Offenders: An Integrated Approach.

[42] Ward T, Brown M (2004). The good lives model and conceptual issues in offender rehabilitation. Psychol Crime Law.

[43] National Institute for Health and Care Excellence (NICE). Drug misuse in over 16s: psychosocial interventions: NICE Guideline [CG51]. 2007. https://www.nice.org.uk/guidance/cg51/chapter/Appendix-C-Contingency-management-key-elements-in-the-delivery-of-a-programme. Accessed 10 Apr 2019. 31877006

[44] Murphy CM, Ting L (2010). The effects of treatment for substance use problems on intimate partner violence: a review of empirical data. Aggress Violent Behav.

[45] Babor TF, Higgins-Biddle JC, Saunders JB, Monteiro MG (1992). The alcohol use disorders identification test.

[46] Berman AH, Bergman H, Palmstierna T, Schlyter F (2005). Evaluation of the drug use disorders identification test (DUDIT) in criminal justice and detoxification settings and in a Swedish population sample. Eur Addict Res.

[47] Marsden J, Farrell M, Bradbury C, Dale-Perera A, Eastwood B, Roxburgh M, Taylor S (2008). Development of the treatment outcomes profile. Addiction..

[48] McLellan AT, Luborsky L, Woody GE, O'Brien CP (1980). An improved diagnostic evaluation instrument for substance abuse patients. The Addiction Severity Index. J Nerv Ment Dis.

[49] Kroenke K, Spitzer RL, Williams JB (2001). The PHQ-9: validity of a brief depression severity measure. J Gen Intern Med.

[50] Spitzer RL, Kroenke K, Williams JB, Löwe B (2006). A brief measure for assessing generalized anxiety disorder: the GAD-7. Arch Intern Med.

[51] Prins A, Bovin MJ, Smolenski DJ, Marx BP, Kimerling R, Jenkins-Guarnieri MA, Kaloupek DG, Schnurr PP, Kaiser AP, Leyva YE, Tiet QQ (2016). The primary care PTSD screen for DSM-5 (PC-PTSD-5): development and evaluation within a veteran primary care sample. J Gen Intern Med.

[52] Moran P, Leese M, Lee T, Walters P, Thornicroft G, Mann A (2003). Standardised Assessment of Personality–Abbreviated Scale (SAPAS): preliminary validation of a brief screen for personality disorder. Br J Psychiatry.

[53] Graham-Kevan N, Archer J (2005). Investigating three explanations of women's relationship aggression. Psychol Women Q.

[54] Heavey CL, Larson BM, Zumtobel DC, Christensen A (1996). The Communication Patterns Questionnaire: the reliability and validity of a constructive communication subscale. J Marriage Fam.

[55] Lila M, Oliver A, Catalá-Miñana A, Galiana L, Gracia E (2014). The intimate partner violence responsibility attribution scale (IPVRAS). Eur J Psychol Appl L.

[56] Dutton DG (1995). A scale for measuring propensity for abusiveness. J Fam Violence.

[57] Woodlock D (2017). The abuse of technology in domestic violence and stalking. Violence against Women.

[58] Tangney JP, Baumeister RF, Boone AL (2004). High self-control predicts good adjustment, less pathology, better grades, and interpersonal success. J Pers.

[59] Al-Janabi H, Flynn TN, Coast J (2012). Development of a self-report measure of capability wellbeing for adults: the ICECAP-A. Qual Life Res.

[60] Brooks R, Group E (1996). EuroQol: the current state of play. Health Policy.

[61] Dolan P (1997). Modeling valuations for EuroQol health states. Med Care.

[62] Munder T, Wilmers F, Leonhart R, Linster HW, Barth J (2010). Working Alliance Inventory-Short Revised (WAI-SR): psychometric properties in outpatients and inpatients. Clinical Psychol Psychother.

[63] Gaston L (1991). Reliability and criterion-related validity of the California Psychotherapy Alliance Scales—patient version. Psychol Assess.

[64] Felitti VJ (2009). Adverse childhood experiences and adult health. Acad Pediatr.

[65] Paulhus DL, Robinson JP, Shaver PR, Wrightsman LS (1991). Measurement and control of response bias. Measures of personality and social psychological attitudes.

[66] Levesque DA, Gelles RJ, Velicer WF (2000). Development and validation of a stages of change measure for men in batterer treatment. Cognit Ther Res.

[67] World Medical Association Declaration of Helsinki (2013). Ethical principles for medical research involving human subjects. 1. JAMA.

[68] Sim J, Lewis M (2012). The size of a pilot study for a clinical trial should be calculated in relation to considerations of precision and efficiency. J Clin Epidemiol.

[69] Ritchie J, Lewis J, Nicholls CM, Ormston R (2013). Qualitative research practice: A guide for social science students and researchers.

[70] Vos T, Astbury J, Piers LS, Magnus A, Heenan M, Stanley L, Walker L, Webster K (2006). Measuring the impact of intimate partner violence on the health of women in Victoria, Australia. Bull World Health Organ.

[71] Oliver R, Alexander B, Roe S, Wlasny M. The economic and social costs of domestic abuse Research Report 107. Home Office: 2019 https://assets.publishing.service.gov.uk/government/uploads/system/uploads/attachment_data/file/772180/horr107.pdf. Accessed 10 Apr 2019.

