# Supplementary material for: A study protocol to assess the feasibility of conducting an evaluation trial of the ADVANCE integrated intervention to address both substance use and intimate partner abuse perpetration to men in substance use treatment
Source: Pilot Feasibility Stud. 2020 May 11;6:62. doi: 10.1186/s40814-020-00580-7 (PMC7212681; doi:10.1186/s40814-020-00580-7)
Supplement: Supplementary file 1 — Additional file 1: Table S3. The TIDieR (Template for Intervention Description and Replication) Checklist. Information to include when describing an intervention and the location of the information. [file 40814_2020_580_MOESM1_ESM.docx]

**Additional file 1: Table S3. The TIDieR (Template for Intervention Description and Replication) Checklist**

**
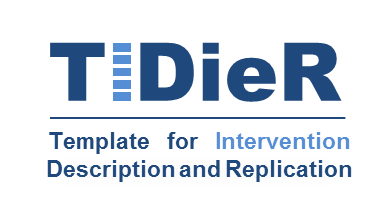
The TIDieR (Template for Intervention Description and Replication) Checklist*:**

Information to include when describing an intervention and the location of the information

| **Item number** | **Item** | **Where located **** | |
| --- | --- | --- | --- |
|  |  | Primary paper  (page or appendix  number) | Other ^†^ (details) |
|  | **BRIEF NAME** |  |  |
| **1.** | Provide the name or a phrase that describes the intervention. | ‘Design’ - Methods section |  |
|  | **WHY** |  |  |
| **2.** | Describe any rationale, theory, or goal of the elements essential to the intervention. | ‘Intervention arm’ - Methods section |  |
|  | **WHAT** |  |  |
| **3.** | Materials: Describe any physical or informational materials used in the intervention, including those provided to participants or used in intervention delivery or in training of intervention providers. Provide information on where the materials can be accessed (e.g. online appendix, URL). | ‘Intervention arm’ – Methods section |  |
| **4.** | Procedures: Describe each of the procedures, activities, and/or processes used in the intervention, including any enabling or support activities. | ‘Inclusion and exclusion criteria, Recruitment, Follow-up, Methods of Randomisation, Blinding’- Methods section | _____________ |
|  | **WHO PROVIDED** |  |  |
| **5.** | For each category of intervention provider (e.g. psychologist, nursing assistant), describe their expertise, background and any specific training given. | ‘Intervention arm’ - Methods section | _____________ |
|  | **HOW** |  |  |
| **6.** | Describe the modes of delivery (e.g. face-to-face or by some other mechanism, such as internet or telephone) of the intervention and whether it was provided individually or in a group. | ‘Intervention arm’ - Methods section | _____________ |
|  | **WHERE** |  |  |
| **7.** | Describe the type(s) of location(s) where the intervention occurred, including any necessary infrastructure or relevant features. | ‘Intervention arm’ - Methods section |  |
|  | **WHEN and HOW MUCH** |  |  |
| **8.** | Describe the number of times the intervention was delivered and over what period of time including the number of sessions, their schedule, and their duration, intensity or dose. | ‘Intervention arm’ - Methods section | _____________ |
|  | **TAILORING** |  |  |
| **9.** | If the intervention was planned to be personalised, titrated or adapted, then describe what, why, when, and how. | N/A | _____________ |
|  | **MODIFICATIONS** |  |  |
| **10.^ǂ^** | If the intervention was modified during the course of the study, describe the changes (what, why, when, and how). | N/A | _____________ |
|  | **HOW WELL** |  |  |
| **11.** | Planned: If intervention adherence or fidelity was assessed, describe how and by whom, and if any strategies were used to maintain or improve fidelity, describe them. | ‘Fidelity’ - Methods section | _____________ |
| **12.^ǂ^** | Actual: If intervention adherence or fidelity was assessed, describe the extent to which the intervention was delivered as planned. | N/A | _____________ |
